# Anti-epileptic drug harms: issues for meta-analysis

**DOI:** 10.1186/1745-6215-12-S1-A11

**Published:** 2011-12-13

**Authors:** Catrin Tudur Smith, Arif Shukralla, Sarah Donegan, Karla Hemming, Graham A Powell, Paula Williamson, Anthony Marson

**Affiliations:** 1 North West Hub for Trials Methodology Research, Department of Biostatistics, University of Liverpool, Liverpool, UK; 2 Clinical and Molecular Pharmacology, University of Liverpool, Liverpool, UK; 3 Public Health, Epidemiology and Biostatistics, University of Birmingham, Birmingham, UK

## Objectives

Decisions regarding choice and dose of anti-epileptic drug (AED) are driven by considering the potential benefits of reducing seizure frequency against the potential harms of alternative AEDs. Such decisions should be made using the best available evidence, which often requires a quantitative synthesis of data from multiple randomised controlled trials (RCT). However, the systematic review and meta-analysis of harms data is hindered by problems such as inadequate reporting, heterogeneity of harms definitions, and selective reporting bias. Here we will evaluate the quality of reporting of harms data in epilepsy trials, and assess the potential added value of incorporating harms data beyond the clinical indication of epilepsy.

## Methods

To evaluate the quality of reporting of harms data in RCTs of AEDs in patients with epilepsy we have undertaken a systematic review [1]. We searched MEDLINE, the Cochrane Library and the Epilepsy Group register for published trials comparing AEDs in patients with epilepsy. Each trial was assessed according to a 23 item checklist developed from the CONSORT statement for the reporting of harms in clinical trials [2]. In a separate analysis, Bayesian panoramic meta-analysis models [3] were used to pool estimates of harm across studies and across indications of epilepsy, neuropathy and headache, allowing for variation between both study and indication.

## Results

For the reporting quality review we identified 152 RCTs that met the eligibility criteria. None of the trials satisfied all criteria. The mean number of criteria per trial was 11.3 (standard deviation 4.3, range 0 to 21). No improvement could be detected following publication of the CONSORT statement for harms (difference in means: 0.6 with 95% CI (-0.9 to 1.8) p=0.53). Items that were not frequently reported were; definition of adverse events (36.2% of trials), use of a validated dictionary (21.7% of trials), use of a validated instrument (15.8% of trials), reporting of both number of patients and number of adverse events (19.1% of trials) and methods for handling of recurrent events (7.2% of trials). In the summary of harms data, borrowing strength from other indications resulted in a more precise effect estimate, and indicate that there is evidence for some adverse events across the range of indications.

## Conclusion

Reporting of harms in RCTs of AEDs is poor and has not improved since the publication of the CONSORT guidelines on the reporting of harms. To allow reliable meta-analyses of harms data, improvements to reporting quality are essential. Preliminary results suggest that harms data from AEDs prescribed for headache and neuropathy may be useful to inform the harms profile of AEDs prescribed for epilepsy.
